# Adverse effect of increased left ventricular wall thickness on five year outcomes of patients with negative dobutamine stress

**DOI:** 10.1186/1532-429X-11-25

**Published:** 2009-08-03

**Authors:** Thomas F Walsh, Erica Dall'Armellina, Haroon Chughtai, Timothy M Morgan, William Ntim, Kerry M Link, Craig A Hamilton, Dalane W Kitzman, W Gregory Hundley

**Affiliations:** 1 Department of Internal Medicine (Cardiology Section), Wake Forest University School of Medicine, Medical Center Boulevard, Winston-Salem, North Carolina, USA; 2 Division of Public Health Sciences, Wake Forest University School of Medicine, Medical Center Boulevard, Winston-Salem, North Carolina, USA; 3 Department of Biomedical Engineering, Wake Forest University School of Medicine, Medical Center Boulevard, Winston-Salem, North Carolina, USA; 4 Department of Radiology, Wake Forest University School of Medicine, Medical Center Boulevard, Winston-Salem, North Carolina, USA

## Abstract

**Background:**

To determine if patients without dobutamine induced left ventricular wall motion abnormalities (WMA) but an increased LV end-diastolic wall thickness (EDWT) exhibit a favorable cardiac prognosis.

**Results:**

Between 1999 and 2001, 175 patients underwent a dobutamine stress cardiovascular magnetic resonance (DCMR) procedure utilizing gradient-echo cines. Participants had a LV ejection fraction >55% without evidence of an inducible WMA during peak dobutamine/atropine stress. After an average of 5.5 years, all participants were contacted and medical records were reviewed to determine the post-DCMR occurrence of cardiac death, myocardial infarction (MI), and unstable angina (USA) or congestive heart failure (CHF) warranting hospitalization.

In a multivariate analysis, that took into account Framingham and other risk factors associated with cardiac events, a cine gradient-echo derived LV EDWT ≥12 mm was associated independently with an increase in cardiac death and MI (HR 6.0, p = 0.0016), and the combined end point of MI, cardiac death, and USA or CHF warranting hospitalization (HR 3.0, p = 0.0005).

**Conclusion:**

Similar to echocardiography, CMR measures of increased LV wall thickness should be considered a risk factor for cardiac events in individuals receiving negative reports of inducible ischemia after dobutamine stress. Additional prognostic studies of the importance of LV wall thickness and mass measured with steady-state free precession techniques are warranted.

## Background

Left ventricular (LV) wall motion abnormalities (WMA) induced during intravenous dobutamine are associated with flow limiting epicardial coronary artery stenoses, and predict future cardiac events including myocardial infarction (MI) and cardiac death [1-4]. Typically, if resting LV end-diastolic wall thickness (EDWT) is normal, the absence of dobutamine inducible WMA identifies a group of individuals with a low risk of experiencing future cardiac events [5,6]. Importantly however, in individuals with increased resting LV EDWT, the sensitivity of dobutamine induced LV WMA for identifying flow limiting epicardial stenoses is low (36%) [7]. Also, data from Framingham have shown that increased LV EDWT itself is an independent predictor of cardiac events [8]. To date, it remains uncertain whether individuals with increased resting LV EDWT and an absence of inducible LV WMA during intravenous dobutamine remain at a relatively low risk of developing a future cardiac event.

This study was performed to determine if the absence of inducible WMA during intravenous dobutamine would be associated with a favorable cardiac prognosis regardless of resting LV EDWT. To address this question, we measured LV EDWT and performed extensive follow-up on individuals undergoing dobutamine stress cardiovascular magnetic resonance (DCMR) examinations with cine white blood fast-gradient echo techniques.

## Methods

### Population and Study Design

The study complies with the Declaration of Helsinki, and was approved by the Institutional Review Board at the Wake Forest University School of Medicine. All patients gave both verbal (for performing follow-up questionnaire) and written (for CMR and later for review of medical records) informed consent. Between 1997 and 2001, 175 consecutive participants with a LV ejection fraction >55%, and no inducible LV WMA indicative of ischemia in any of 17 myocardial segments during DCMR were enrolled in the study. After DCMR, participants blinded to DCMR test results performed the outcomes analysis.

### Dobutamine/Atropine Cardiovascular Magnetic Resonance

As previously described [3,5], images were obtained on a Horizon 1.5T whole-body imaging system (General Electric Medical Systems) using cine white blood spoiled gradient-echo imaging with a 256 × 128 matrix, a 35-48-cm field of view, a 4-ms echo time, a 10-ms repetition time, a 20-degree flip angle, an 8-mm slice thickness, a 40-ms temporal resolution, and 8 to 12 second periods of breath holding. Each of the patients received atropine if they were unable to obtain 80% maximum predicted heart rate for age (n = 95). Findings of this heart rate response have been shown at our institution to be 83% sensitive and 83% specific for identifying >50% coronary arterial luminal narrowings during dobutamine/atropine stress, and have been shown to forecast future cardiac events [3,5].

**Figure 1:**
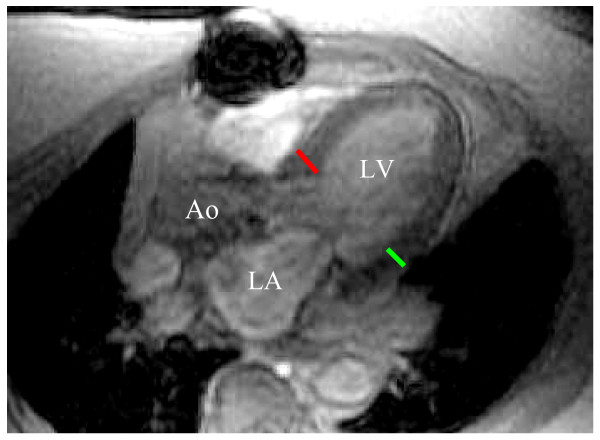
**In accordance with the American Society of Echocardiography, posterior (green line) and septal (red line) left ventricular wall thickness was measured in the left ventricular long axis view at end diastole at approximately the level of the mitral valve leaflet tips (LV = left ventricle; LA = left atrium; and Ao = aorta)**.

At rest and during graded doses of dobutamine/atropine stress, LV wall motion was confirmed as normal across all 17 myocardial segments [5]. The resting LV ejection fraction was measured using a biplane area-length technique [9]. According to previously published techniques, the posterior and septal wall thicknesses were measured at the level of the LV minor dimension, at the mitral chordae level using the end-diastolic, left ventricular 3-chamber (equivalent to transthoracic echocardiography parasternal long-axis) view (Figure 1) [10]. At the time of testing, the occurrence of a prior Q-wave myocardial infarction [11], and the presence of cardiac risk factors [including a history of diabetes [12], smoking, coronary revascularization, elevated total cholesterol [13], and hypertension [14] were recorded.

### Outcomes

Within 7 years of the DCMR exam, each participant was contacted to determine the post-procedure occurrence of cardiac events. All persons were contacted initially by phone or personal interview. If deceased, the next of kin was contacted. Questions were administered to identify the possibility of myocardial infarction (MI), unstable angina (USA) or congestive heart failure (CHF). Any change in physical state, medical condition, or medicationwas confirmed in all cases by review of the participant's medical records. If an event was suspected or identified during contact with the participant, medical records from the site of the event were obtained and confirmation of the event was determined according to data collected from the medical record. Established, published criteria were utilized to define events. Hard events were defined as cardiac death (death in the presenceof acute MI, significant cardiac arrhythmia, or refractory CHF [15], or MI (angina of >30 minutes durationand either ≥ 2 mm ST segment elevation in 2 consecutive ECG leadsor a rise in cardiac enzymes including creatine kinase level MB fraction cardiac troponin-I above normal limits) [16]. Electrocardiographic, enzymatic, or autopsy data were used tosubstantiate cardiac mortality. Any events were defined as hard events and/or USA, or CHF warranting hospital admission. In the case of 2 simultaneouscardiac events, the worst event was chosen (cardiac death>MI> USA>CHF).

### Statistical Analysis

After all records were obtained, a statistical analysis was conducted using SAS 9.1 (SAS Institute, Cary, NC) for Windows. Based on previously published criteria, participants were dichotomized into those with a LV EDWT < or ≥12 mm [15]. In this study, we determined LV EDWT in the septum, posterior wall, and the average of the two. The average LV EDWT of the septal and posterior walls served as the primary outcome measure. All grouped data were expressed as mean ± SD. Independent predictors of events were identified using univariate and multivariate Cox proportional hazards regression models. The risk of a given variable was expressed by a hazard ratio (HR) with corresponding 95% CIs. A variable was considered significant if the null hypothesis of no contribution could be rejected at a probability value of <0.05. The probability of the presence or absence of hard events as a function of follow-up duration was estimated by the Kaplan-Meier method and compared between groups by use of the log-rank test. Unadjusted, Framingham adjusted, and additional cardiac MI risk factor adjusted Cox proportional hazard regression models were used to predict cardiac events.

To determine the intra observer variability for measuring LV EDWT, a randomly selected sample of 20 participants was analyzed twice separated by 1 year. To determine inter observer agreement in measures of LV EDWT, this same sample from 20 participants was analyzed by a separate individual. In both the intra and inter observer comparisons, repeat assessments were performed in an unpaired fashion blinded to the results of prior analyses.

The authors had full access to the data and take responsibility for its integrity.

## Results

Contact was made with all 175 patients; their clinical data are displayed in Table 1. Those with an average (septal and posterior wall) LV EDWT ≥12 mm were older, and exhibited more prior CAD, hypertension, diabetes, and lung disease. Overall, the amount of dobutamine administered was 30 ± 10 μg/kg/min and did not differ between those with and without a LV EDWT < or ≥12 mm. Rest and stress hemodynamic assessments at the time of DCMR stress were similar for those with or without LV EDWT < or ≥12 mm (Table 2).

**Table 1:** Demographic data

			LV EDWT		LV EDWT		*p*-value
		
	Total		≤12 mm		>12 mm		
		
	n = 175		n = 98		n = 77		
Patient Characteristics							
Age (yrs)	69	(± 12)	65	(± 15)	71	(± 10)	*0.013*
Gender Women, Men	102	73.0	63	35.0	39	38.0	*0.07*
Weight (kg)	92.2	(± 23.4)	90.6	(± 23.0)	94.3	(± 23.7)	*0.32*
BSA (m^2^)	2.3	(± 0.7)	2.1	(± 0.7)	2.4	(± 0.8)	*0.56*
Wall Thickness	12.3	(± 3.1)	9.2	(± 0.8)	16.0	(± 2.4)	*0.0001*
							
Historical Information							
Prior CAD	81	(50.0%)	41	(41.8%)	40	(51.9%)	*0.190*
Prior Revascularization	28	(16.0%)	10	(10.2%)	18	(23.4%)	*0.018*
Hypertension	112	(64.0%)	52	(53.1%)	60	(77.9%)	*0.0006*
Diabetes Melitus	51	(29.1%)	23	(23.5%)	28	(36.4%)	*0.063*
Hyperlipidemia	89	(50.9%)	46	(46.9%)	43	(55.8%)	*0.240*
Smoking	64	(36.6%)	31	(31.6%)	33	(42.9%)	*0.130*
Asthma/COPD	39	(22.3%)	16	(16.3%)	23	(29.9%)	*0.033*
							
Medications							
Digoxin	9	(5.1%)	6	(6.1%)	3	(3.9%)	*0.51*
Vasodilator	14	(8.0%)	7	(7.1%)	28	(36.4%)	*0.64*
Diuretic	60	(34.3%)	32	(32.7%)	28	(36.4%)	*0.61*
Beta Blocker	54	(30.9%)	23	(23.5%)	31	(40.3%)	*0.017*
Calcium Antagonist	32	(18.3%)	13	(13.3%)	19	(24.7%)	*0.053*
ASA	82	(46.9%)	43	(43.9%)	39	(50.6%)	*0.38*
Nitrate	45	(25.7%)	20	(20.4%)	25	(32.5%)	*0.071*
ACE Inhibitor	39	(22.3%)	16	(16.3%)	23	(29.9%)	*0.033*
Anti-Coagulation	15	(8.6%)	4	(4.1%)	11	(14.3%)	*0.017*
Statin	65	(37.1%)	28	(28.6%)	37	(48.1%)	*0.0079*

Abbreviations: ACE, angiotensin converting enzyme; ASA, aspirin, BSA; body surface area; CAD, coronary artery disease; CHF, congestive heart failure; COPD, chronic obstructive airways disease, LV EDWT, left ventricular end-diastolic wall thickness.

**Table 2:** Hemodynamic data

Hemodynamic Data	Total	LV EDWT	LV EDWT	*p*-*v*alue
		
	≤12 mm		>12 mm	
		
	Rest			
Heart Rate (bpm)	73 ± 18	73 ± 17	73 ± 20	0.99
Systolic Blood Pressure (mmHg)	136 ± 38	134 ± 35	138 ± 43	0.48
Diastolic Blood Pressure (mmHg)	76 ± 22	75 ± 20	77 ± 25	0.48

	Stress			

Heart Rate (bpm)	129 ± 28	131 ± 30	126 ± 27	0.30
Systolic Blood Pressure (mmHg)	143 ± 42	143 ± 40	143 ± 47	0.93
Diastolic Blood Pressure (mmHg)	74 ± 24	73 ± 21	76 ± 27	0.59

Abbreviations: bpm, blood pressure measure; mmHg, millimeters of mercury; LV EDWT, left ventricular end-diastolic wall thickness.

Over the 5.5 average years of follow-up, the rate of hard events was 8.4% (Table 3). The 5.5 year hard event rate was 3.2% for the patients with a LV EDWT <12 mm compared to 20.3% for those with a LV EDWT ≥12 mm. The EDWT for the posterior wall averaged 12 ± 3 mm in participants with no events, and 14 ± 3 mm in participants with any events (p < 0.001). Similarly, the septal wall thickness in patients without events was 12 ± 3 mm compared to 14 ± 4 mm for those with any events (p < 0.001). The septal and posterior wall thickness among the participants were highly correlated (r = 0.76, p = 0.001). There was no difference in the predictive accuracy of the septal, posterior, or combined average of the wall thickness for identifying those at risk of future cardiac events.

**Table 3:** Table of events

		Total	LV EDWT		LV EDWT		*p*-value
		
			≤12 mm		>12 mm		
Hard Events	16	(8.4%)	3	(3.2%)	13	(20.3%)	*0.0015*
Cardiac Deaths	12	(6.4%)	3	(3.2%)	9	(13.2%)	*0.025*
Myocardial Infarction	4	(2.2%)	0	(0.0%)	4	(5.5%)	*0.022*
							
Non Hard Event Hospital Admissions	26	(12%)	12	(12.2%)	14	(22.2%)	*0.37*
Congestive Heart Failure	2	(1.1%)	0	(0.0%)	2	(2.7%)	*0.11*
Unstable-Angina	24	(13.1%)	12	(12.2%)	12	(15.6%)	*0.4*

Abbreviation: LV EDWT, left ventricular end-diastolic wall thickness

**Figure 2:**
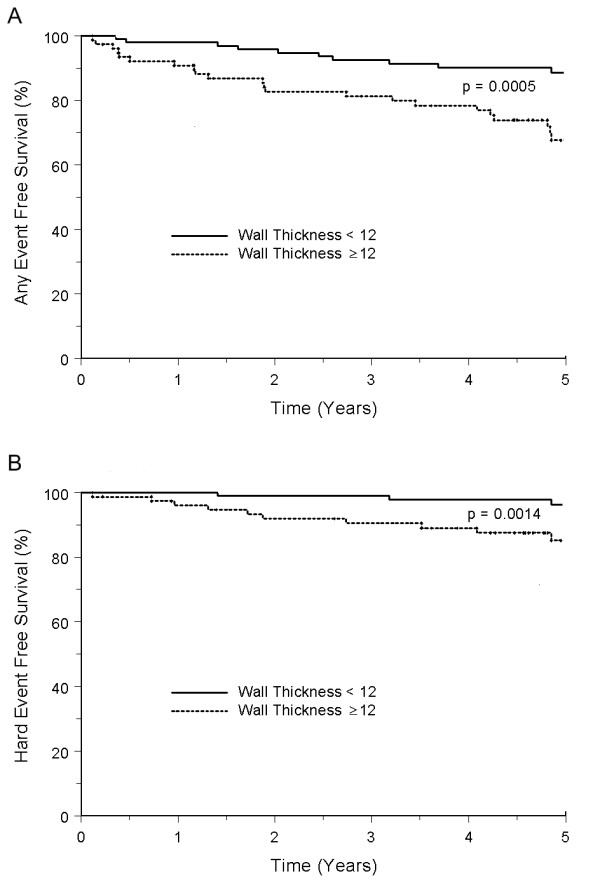
**Kaplan-Meier survival curves for participants free from any cardiac event (Panel A), or a hard cardiac event (Panel B)**. Graphs for individuals with and without LV end-diastolic wall thickness >12 mm are shown.

**Figure 3:**
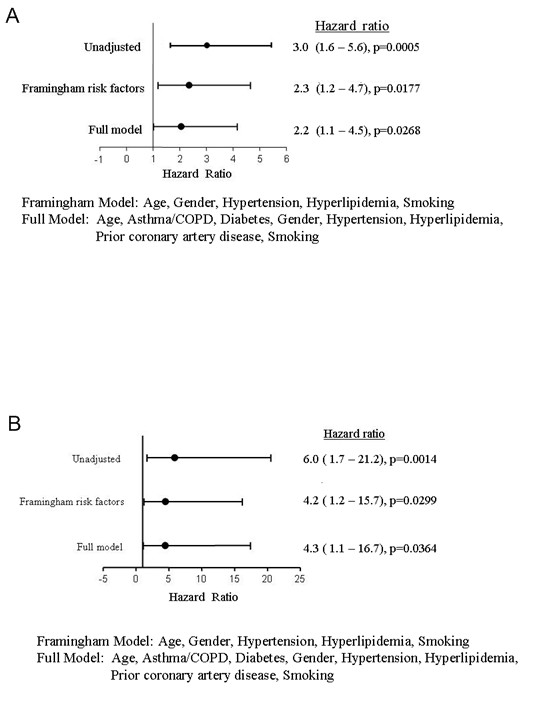
**Hazard ratios from multivariate analyses for average LV end-diastolic wall thickness >12 mm for any (Panel A) and hard (Panel B) cardiac events**. As shown, the top row represents the unadjusted Cox proportional hazard model, the second row represents the Cox proportional hazard model adjusted for Framingham risk factors, and the last row represents the Cox proportional hazard model adjusted for Framingham risk factors and other factors associated with cardiac events.

The proportion of participants free of both hard and any cardiac events is shown in Figure 2. A LV EDWT ≥12 mm was predictive of both any and hard events in a) the unadjusted model, b) the Framingham risk factor adjusted model, and c) after adjustment for factors associated with CAD, MI, and cardiac events (Figure 3 and Table 4). We also performed analyses treating LV EDWT as a continuous, as opposed to dichotomous (< or ≥12 mm thick) variable. Using the proportional hazard wall thickness as a continuous variable demonstrated a significant trend of increasing risk of hard events (p = 0.004) and any events (p = 0.001) with increasing LV EDWT.

**Table 4:** Multivariate predictors of events expressed as hazard ratio (± 95% confidence intervals)

	**Multivariate**	
**Covariate**	**CV Death/MI**	**Any Event**
Age (Years)	1.00 (0.97 - 1.03)	1.05 (0.99 - 1.10)
Hypertension	1.32 (0.60 - 2.90)	0.64 (0.17 - 2.35)
Receiving Statin	1.14 (0.58 - 2.25)	0.56 (0.19 - 1.66)
Diabetes	1.79 (0.94 - 3.49)	2.99 (0.94 - 9.56)
Male Gender	1.31 (0.69 - 2.48)	2.88 (0.85 - 9.72)
Prior CAD or Revasc.	1.74 (0.90 - 3.37)	1.58 (0.54 - 4.62)
Smoking	1.19 (0.63 - 2.25)	1.33 (0.47 - 3.77)
Wall Thickness ≥ 12	2.34 (1.15 - 4.74)	4.28 (1.10-16.62)

Abbreviations: CAD, coronary artery disease; Revasc., prior coronary artery revascularization procedure.

The intra observer correlation of LV EDWT was 0.73, and the inter observer assessment of LV EDWT was 0.77.

## Discussion

Prior studies have found that increased LV EDWT during a dobutamine stress test is associated with a decreased sensitivity for detecting flow limiting coronary arterial luminal narrowings detected with contrast coronary angiography [7]. Other studies have identified an association between the presence of LV hypertrophy or increased LV EDWT and an adverse cardiac prognosis [8,17-19]. In many countries, dobutamine stress echocardiography is performed widely for individuals suspected to have or possessing coronary arteriosclerosis, MI, or CHF. Individuals without inducible LV WMA are ascribed a favorable prognosis, and thus often are not referred for aggressive risk management. Given the prevalence of risk factors such as hypertension, and an abnormal increase in LV EDWT in these patients, we felt it important to determine if the favorable prognosis in patients with a negative dobutamine stress test and a normal resting LV EDWT would occur in patients with a negative dobutamine stress test and an increase in resting LV EDWT. Our results indicate that in the absence of inducible LV WMA during intravenous dobutamine, either an average, a septal, or a posterior LV EDWT ≥12 mm measured in the 3-chamber (similar to echocardiographic parasternal long-axis view) using gradient-echo techniques was associated independently with future adverse cardiovascular events (Figure 2).

Four possibilities could explain our results. First, with dobutamine stress echocardiography, previous investigators have noted an inability to visualize all myocardial wall segments during cardiac stress testing procedures [3,5], and thus one potentially can miss visualization of an inducible LV wall motion abnormality indicative of ischemia (a known risk factor for cardiac events) during intravenous dobutamine. For this reason, we utilized CMR because of previously reported high image quality and ability to assess both LV EDWT and WMA throughout the course of a dobutamine infusion [3-6]. In the current study, LV wall motion was visualized throughout the course of testing in all segments for all participants; and thus, inadvertently missing a stress induced LV wall motion abnormality due to suboptimal image quality is not felt to be the cause of the poor prognosis observed within the participants of the current study.

Second, as shown in Table 1, the participants that experienced an increased incidence of cardiovascular events also exhibited many illnesses or clinical conditions, including hypertension, advanced age, prior coronary artery disease and diabetes, that have been associated previously with an adverse cardiac prognosis [11-14]. Importantly however, after serial adjustments utilizing multivariate models that included Framingham risk factors (age, gender, smoking, hypertension, elevated cholesterol) and other clinical conditions associated with adverse cardiac events, the presence of a LV EDWT ≥12 mm was a predictor of an adverse cardiac prognosis independent of the association that our participants may have had with known risk factors for adverse cardiac events. Thus, these analyses support the notion that LV EDWT is an independent risk factor, and that the poor prognosis identified in our participants was not due to confounding from a variable already known to be associated with adverse cardiovascular risk.

Third, Smart, et al [7], have shown that in individuals with increased LV EDWT, the utility of dobutamine induced WMA for identifying inducible ischemia in patients with single vessel coronary artery disease is substantially reduced [7]. This is thought secondary to enhanced epicardial contraction in the thickened wall that can overcome the loss of contraction that may occur as endocardial tissue becomes ischemic during stress [7]. It may be that our participants with increased LV EDWT exhibited myocardial ischemia in endocardial regions due to undiagnosed CAD. In turn, this unidentified inducible ischemia could have accounted for their future cardiac events. Addressing this point could occur with gadolinium enhanced first-pass dobutamine perfusion techniques [20].

Fourth, the presence of increased LV EDWT may portend a poor prognosis independent of the presence of coronary arteriosclerosis. Increases in LV EDWT can result from one or more of several factors including: increased LV afterload (due in part to increased vascular stiffness, factors influencing the neuro-hormonal axis (for example elevations of renin, angiotensin, or aldosterone), or preexisting genetic abnormalities [21,22]. Also, preclinical hypertrophic cardiomyopathy has been described [23]. Each of these variables in isolation or in combination with the other variables are associated with adverse cardiac events. In addition, their influence on the left ventricle promotes myocyte hypertrophy (often manifest as increased wall thickness) which is also an independent predictor of cardiac events [24]. Since we did not measure factors influencing the neuro-hormonal axis or assess genetic factors in the current study, we cannot comment on the potential influence of these variables on our outcomes.

The findings of the current study have several important clinical implications for the management of patients with chest pain syndromes. First, the absence of dobutamine induced WMA in individuals with a resting left ventricular EDWT of ≥12 mm does not forecast the same cardiac prognosis as for individuals with a LV EDWT <12 mm. In fact, in the participants in this study with a LVEF >55% and no inducible LV WMA, the 2-year hard event free survival was 89%. In a previous study from our group that included patients referred for dobutamine stress that exhibited inducible ischemia and a LVEF >40%, the 2-year event free survival was 84% [5]. In short, in the setting of resting LV EDWT ≥12 mm and no inducible WMA, one should be concerned about not only the presence of undetected CAD (as noted by Smart, et al.)[7], but also that these individuals may have a poor cardiac prognosis relative to individuals with a wall thickness <12 mm.

Second, further investigation should be considered to risk stratify patients with a LV EDWT >12 mm but without inducible WMA during intravenous dobutamine. Recently, Paetsch, et al [20], has demonstrated the utility of vasodilator first pass perfusion imaging in order to identify perfusion defects at the endocardial level associated with epicardial coronary artery stenoses. Additionally, in the setting of patients with hypertrophied ventricles or underlying CAD, Kwong et al.[25], and Moon et al [26], have shown recently that the presence of Late gadolinium enhancement is associated with fibrosis and an adverse cardiac prognosis.

Third, it is important to recognize that we did not measure left ventricular mass in the current study. Thus, those individuals with eccentric left ventricular hypertrophy, who may have normal wall thickness, or those with concentric remodeling, who may or may not have an absolute increase in wall thickness depending upon their left ventricular cavity size, are not included in the current analyses. For this reason, these data are somewhat preliminary and further studies that would understand the relationship between left ventricular hypertrophy and the patterns of hypertrophy and adverse cardiovascular events are warranted.

Fourth, the study was performed using measures of LV EDWT derived from cine, gradient-echo white blood imaging techniques. At 1.5 T, this image acquisition strategy often exhibits flow artifacts along the LV endocardial surface, particularly in apical views when LV systolic function is reduced. Though this technique has been used in large 6000 person population studies such as the Multi-Ethnic Study of Atherosclerosis [27], newer steady-state free precession cine white blood imaging techniques are more frequently used clinically. These data suggest similar studies should be performed that determine the prognostic importance of routine measures of heart size using steady-state free precession techniques.

Our study has the following limitations. First, according to previously published criteria from transthoracic echocardiography, we selected a dichotomous variable of < or ≥12 mm in LV EDWT as our primary outcome variable [10]. Abnormal measures of LV EDWT have not been well established for gradient-echo or steady-state free precession CMR. These data suggest that future studies, similar to those performed with echocardiography, are warranted to establish prognostic importance of routinely acquired CMR parameters of wall thickness. Although, the sample size was not large enough to have good power to detect moderate size effects, the observed hazard ratios of 6.0 for hard events and 3.0 for any events were large enough to demonstrate that there is a statistically (and clinically) significant increased risk of hard or any events with increased wall thickness.

Second, measures of LV mass using a multi-slice short axis Simpson's Rule acquisition strategy were not acquired in the current study. Importantly however, our measures of LV EDWT are easily acquired and can be reported during echocardiographic as well as CMR techniques; hence the clinical applicability of this finding is high. Finally, we are unable to identify whether there is a threshold of LV EDWT that confers information regarding cardiac prognosis among individuals with different race [28]. The precision of the magnetic resonance data combined with the perfect longitudinal follow-up over 7 years allowed us to draw conclusions from a relatively small sample size, but the relevance of this measure across individuals of different race requires further study.

## Conclusion

These data indicate that in those with a LV ejection fraction >55% at rest and no inducible WMA during intravenous dobutamine, a LV EDWT measurement of ≥12 mm using cine white blood gradient-echo imaging techniques is associated with adverse cardiac events. Increased LV wall thickness should be considered a risk factor for cardiac events in individuals receiving negative reports of inducible ischemia after dobutamine stress. Additional prognostic studies of the importance of LV wall thickness and mass measured with steady-state free precession techniques are warranted.

## Abbreviations

CAD: coronary heart disease; CHF: congestive heart failure; DCMR: dobutamine cardiovascular magnetic resonance; EDWT: end-diastolic wall thickness; HR: hazard ratio; LV: left ventricular; MI: myocardial infarction; USA: unstable angina; WMA: wall motion abnormalities.

## Competing interests

The authors declare that funding for MRI image acquistions were in part supported by NIH R01HL074330 and NIH General Clinical Research Center M01RR07122 grants and through a small ownership in Prova, Inc., a company that produces and sells software for cardiac MRI image display.

## Authors' contributions

TFW performed follow-up, designed study and wrote manuscript; EDA performed follow-up, designed study, and wrote manuscript; TMM designed study, provided statistical analysis and edited manuscript; WN designed study and edited manuscript; KML performed MRI studies and image analysis, and edited manuscript; CAH enabled image acquisition and analysis, and edited manuscript; DWK designed study and edited manuscript; WGH designed study, performed MRI studies and image analysis, and edited manuscript.
